# Optimization of a Fluorescence-Based Assay for Large-Scale Drug Screening against *Babesia* and *Theileria* Parasites

**DOI:** 10.1371/journal.pone.0125276

**Published:** 2015-04-27

**Authors:** Mohamed Abdo Rizk, Shimaa Abd El-Salam El-Sayed, Mohamed Alaa Terkawi, Mohamed Ahmed Youssef, El Said El Shirbini El Said, Gehad Elsayed, Sabry El-Khodery, Maged El-Ashker, Ahmed Elsify, Mosaab Omar, Akram Salama, Naoaki Yokoyama, Ikuo Igarashi

**Affiliations:** 1 National Research Center for Protozoan Diseases, Obihiro University of Agriculture and Veterinary Medicine, Inada-Cho, Obihiro, Hokkaido, Japan; 2 Department of Internal Medicine and Infectious Diseases, Faculty of Veterinary Medicine, Mansoura University, Mansoura, Egypt; 3 Department of Biochemistry and Chemistry of Nutrition, Faculty of Veterinary Medicine, Mansoura University, Mansoura, Egypt; 4 Department of Animal Medicine and Infectious Diseases, Faculty of Veterinary Medicine, Minoufiya University, Sadat City, Minoufiya, Egypt; 5 Department of Parasitology, Faculty of Veterinary Medicine, South Valley University, Qena, Egypt

## Abstract

A rapid and accurate assay for evaluating antibabesial drugs on a large scale is required for the discovery of novel chemotherapeutic agents against *Babesia* parasites. In the current study, we evaluated the usefulness of a fluorescence-based assay for determining the efficacies of antibabesial compounds against bovine and equine hemoparasites in *in vitro* cultures. Three different hematocrits (HCTs; 2.5%, 5%, and 10%) were used without daily replacement of the medium. The results of a high-throughput screening assay revealed that the best HCT was 2.5% for bovine *Babesia* parasites and 5% for equine *Babesia* and *Theileria* parasites. The IC_50_ values of diminazene aceturate obtained by fluorescence and microscopy did not differ significantly. Likewise, the IC_50_ values of luteolin, pyronaridine tetraphosphate, nimbolide, gedunin, and enoxacin did not differ between the two methods. In conclusion, our fluorescence-based assay uses low HCT and does not require daily replacement of culture medium, making it highly suitable for *in vitro* large-scale drug screening against *Babesia* and *Theileria* parasites that infect cattle and horses.

## Introduction

Babesiosis is a tick-transmitted disease caused by hemoprotozoan parasites and known to bring great economic losses in the bovine and equine industries worldwide. Bovine babesiosis caused by *B*. *bovis* and *B*. *bigemina* have a considerable impact on cattle health and productivity [1]. Equine piroplasmosis, caused by *T*. *equi* [2] and *B*. *caballi* [3], is considered one of the most important protozoan diseases affecting horses, mules, and donkeys [3]. Clinical manifestations of babesiosis include fever, hemolytic anemia, hemoglobinuria, and sometimes death [1, 4]. To date, chemotherapy by imidocarb dipropionate and diminazene aceturate is the most common strategy for controlling infection in the field [5]. However, concerns about the toxicity and resistance of these drugs have emerged [5]. Therefore, there is an urgent need to develop more effective and safer antibabesial drugs.

Microscopic examination of stained blood smears is the gold standard for direct detection of parasites. However, this method is influenced by the quality of the blood smears as well as the skill and experience of experimentators and is not suitable for large-scale drug screening [6]. A new, alternative strategy that offers accuracy, simplicity, and automatic analysis is absolutely necessary [7]. To that end, a fluorescence-based technique using SYBR Green I (SGI) has been proposed as it provides more reliable results in a short time without being influenced by experimentator variations. In fact, this assay has been employed to evaluate the efficacies of antimalarials *in vitro* [8]. In brief, the assay relies on the high throughput screening of red blood cells to detect parasite DNA using a fluorescent spectrophotometer [8]. High-throughput screening (HTS) assay is a well-established process for drug discovery in biotech companies [9]. During the last 2 decades, there has been a dramatic increase in the number of available compounds, leading to a fundamental change in the drug discovery process applied in research units. Strikingly, HTS assay has the ability to test 10,000 to 100,000 compounds per day [9]. Therefore, this assay is one suitable approach to mass screening of compounds.

Although we have recently developed a novel fluorescence-based assay in our laboratory for drug evaluations against *B*. *bovis* [10], the assay is not suitable for large-scale screening of drugs due to the need for daily replacement of the medium, which is time consuming and requires great effort. Moreover, the previous assay has been shown to lower coefficients of variation at the maximal signal (% CV_max_) and the minimal signal (% CV_min_) percentages in comparison with the malaria parasites [11]. Therefore, in the present study, we optimized the assay to be suitable for large-scale screening of drugs and evaluated its usefulness in monitoring the *in vitro* growth of *B*. *bovis*, *B*. *bigemina*, *T*. *equi*, and *B*. *caballi* treated with diminazene aceturate, luteolin, pyronaridine tetraphosphate, nimbolide, gedunin, or enoxacin. The study revealed the promising use of our optimized fluorescence based assay for high throughput screening of compounds against *in vitro* growth of *Babesia* and *Theileria* parasites.

## Material and Methods

### Ethics statement

Experiments described in this article were conducted in accordance with the Guiding Principles for the Care and Use of Research Animals promulgated by Obihiro University of Agriculture and Veterinary Medicine, Japan. The protocol was approved by the Committee on the Ethics of Animal Experiments of the Obihiro University of Agriculture and Veterinary Medicine (Permit numbers 26–27).

### Chemical reagents

SYBR Green I (SGI) nucleic acid stain (Lonza, USA; 10,000x) was stored at -20°C and thawed before use. A lysis buffer consisting of Tris (130 mM; pH 7.5), EDTA (10 mM), saponin (0.016%; W/V), and TritonX-100 (1.6%; V/V) was prepared in advance and stored at 4°C. Diminazene aceturate (Novartis, Japan), luteolin (Sigma-Aldrich, Japan), pyronaridine tetraphosphate (Sigma-Aldrich), nimbolide (BioVision, USA), gedunin (Tocris Bioscience, UK), and enoxacin (Sigma-Aldrich) were prepared as 100 mM or 50 mM stock solutions.

### *In vitro* cultures of *Babesia* parasites

A Texas strain of *B*. *bovis* [12], an Argentina strain of *B*. *bigemina* [13], a U.S. Department of Agriculture (USDA) strain of *B*. *caballi* [14], and a USDA strain of *T*. *equi* (*B*. *equi*) [15] were cultivated in purified bovine or equine red blood cells (RBCs) using a microaerophilic, stationary-phase culture system [16, 17]. Briefly, Medium 199 was used for *B*. *bovis*, *B*. *bigemina*, and *T*. *equi* whereas RPMI 1640 was used for *B*. *caballi* (both from Sigma-Aldrich, Japan). Media were supplemented with 40% normal bovine serum (for bovine *Babesia* isolates) or 40% normal horse serum (for equine *Babesia* and *Theileria* isolates), 60 U/ml penicillin G, 60 μg/ml streptomycin, and 0.15 μg/ml amphotericin B (all three drugs from Sigma-Aldrich). Additionally, 13.6 μg of hypoxanthine (ICN Biomedicals, Inc., USA) per ml was added to *T*. *equi* culture as a vital supplement. Cultures of parasitized RBCs (pRBCs) were incubated at 37°C in an atmosphere of 5% CO2, 5% O2, and 90% N2. After 4 days of culture, the percentages of infected RBCs reached 9.6 to 11.2% for *B*. *bovis*, 5.1 to 6.7% for *B*. *bigemina*, 8.1 to 15.0% for *T*. *equi*, and 6.5 to 8.7% for *B*. *caballi*.

### Assessment of SYBR Green I fluorescence linearity

A 96-well plate was used to assess the linearity between the fluorescent values and parasitemia values as determined by microscopy. Briefly, *B*. *bovis*, *B*. *bigemina*, *T*. *equi*, and *B*. *caballi* pRBCs were serially diluted with non-parasitized bovine or equine RBCs to parasitemia ranging from 0.25% to 8% in 100 uL [18]. Non-parasitized bovine or equine RBCs were used as a blank control. Bovine RBCs were prepared in M199 medium; equine RBCs were prepared in M199 medium for *T*. *equi* and RPMI 1640 medium for *B*. *caballi* in triplicate at three different hematocrit (HCT) (2.5%, 5%, and 10%). One hundred μl of a lysis buffer was mixed with a 2× SGI (10,000x) nucleic acid stain and added directly to each dilution by gentle mixing. Plates were incubated for 24 hours in a dark place at room temperature, and fluorescence values determined using a fluorescence plate reader (Fluoroskan Ascent, Thermo Labsystems, USA) at 485 nm and 518 nm excitation and emission wavelengths, respectively. Gain values were set to 100. The Parasitemia values were plotted against RFU values, after substraction of non-parasitized bovine or equine RBCs, and analyzed by linear regression.

### Determination of statistical parameters for high-throughput screening (HTS) assays

*B*. *bovis*, *B*. *bigemina*, *T*. *equi*, and *B*. *caballi* pRBCs at 1% parasitemia were cultivated in 96-well plates with non-parasitized bovine or equine RBCs at three different HCT percentages (2.5%, 5%, and 10%). Culture medium was M199 or RPMI 1640 alone (positive growth, 100% growth) or supplemented with a supralethal dose (20 uM) of diminazene aceturate (negative control). Then, the cultures of pRBCs were incubated without daily replacement of the medium, and fluorescence values were determined after the fourth day as previously mentioned. Nine samples were used for each medium in triplicate.

### Determination of assay quality

Statistical analyses to determine assay quality (or Z’) were performed according to [19]. Briefly, the equations for calculating the Z’ factor (Z’), signal to noise (S/N) ratio, coefficient of variation at the maximum signal (% CV_max_, positive control), and coefficient of variation at the minimum signal (% CV_min_, negative control) are as follows: Z’ = 1–[(3σ(+)+3σ(-)/μ(+)-μ(-)], S/N ratio = [μ(+)-μ(-)]/σ(-), % CV_max_ = 100x[σ(+)/μ(+)], and % CV_min_ = 100x[σ(-)/μ(-)], respectively, where μ(+) and σ(+) are the mean and standard deviation of pRBCs (positive control), respectively, and μ(-) and σ(-) are the mean and standard deviation of the uninfected bovine and equine RBCs (negative control), respectively.

### Optimization of the fluorescence-based assay

*B*. *bovis* and *T*. *equi* pRBCs at 1% parasitemia were cultivated in 96-well plates at different HCT values (2.5%, 5%, and 10%) in M199 medium alone or with the indicated concentrations of diminazene aceturate (10, 250, 500, 1000, 5000, and 10000 nM) in 100 μl final volume. Non-parasitized RBCs were loaded into each well in triplicate and used as a blank control. RBC cultures were maintained for 4 days without replacement of the medium. On the fourth day, thin blood smears from each well were prepared and stained with Giemsa stain to be observed under the microscope to calculate parasitemia. Next, 100 μl of a lysis buffer containing 2× SGI nucleic acid stain was directly added to each well by gentle mixing. The plates were then incubated for 24 hours in a dark place at room temperature, and the fluorescence values were determined using a fluorescence plate reader as previously mentioned. Gain values were set to 100. The mean fluorescence values (after background subtraction of uninfected bovine and equine RBCs) were then plotted against the logarithm of drug concentrations. The 50% inhibitory concentration (IC_50_) values were calculated on the fourth day of culture using both the fluorescence-based assay and microscopic examination. In a separate experiment, two 96-well plates were used for the cultivation of *B*. *bovis* and *T*. *equi* pRBCs at 1% parasitemia at 2.5% and 5% HCTs, respectively. The pRBCs were cultivated for 4 days with replacement of the medium in triplicate wells for each concentration of the drug. On the fourth day, IC_50_ values of diminazene aceturate were determined as reported above. Each experiment was repeated three times.

### Antibabesial drug screening by fluorescence-based assay

*B*. *bovis*, *B*. *bigemina*, *T*. *equi*, and *B*. *caballi* pRBCs were cultivated at 1% parasitemia in 96-well plates using 2.5% and 5% HCTs for bovine and equine parasites, respectively, and M199 or RPMI 1640 media alone or with the indicated concentrations of drugs, including diminazene aceturate, luteolin, pyronaridine tetraphosphate, nimbolide, gedunin, and enoxacin. The pRBCs were cultivated for 4 days without replacement of the medium in triplicate wells for each concentration of the drug. Four plates were used in quadruplicate for each drug experiment in bovine *Babesia* parasites and in diminazene aceturate only for equine *Babesia* and *Theileria* parasites, and growth inhibition assays [20] between 1 and 4 days post-drug addition were conducted to evaluate whether the drug-induced growth inhibition is reflected by fluorescence signals in a dose-dependent manner. With other drugs for equine *Babesia* and *Theileria* parasites, only one plate was used in each drug experiment for 50% inhibitory concentration (IC_50_) value calculation on the fourth day of culture after adding lysis buffer containing 2× SGI. The IC_50_ values were calculated on the fourth day of culture using both the fluorescence-based assay and microscopic examination. Each experiment was repeated three times.

### Statistical analysis

Data analysis was performed using GraphPad Prism ver. 5 (GraphPad Software, Inc., USA) using the one-way ANOVA. The IC_50_ values were calculated using GraphPad Prism ver. 5. The mean IC_50_ values by fluorescence assay and microscopic examination for each tested drug were analyzed using an unpaired *t*-test with the GraphPad software available online (http://www.graphpad.com/quickcalcs/ttest1.cfm?Format=SD). *P* < 0.05 was considered to be statistically significant.

## Results

### Assessment of SYBR Green I fluorescence linearity

**Fig 1:**
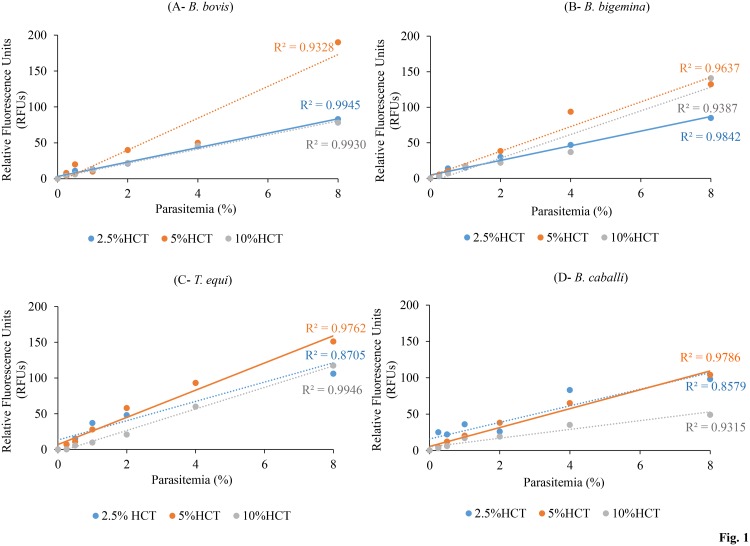
Linearity assessment between relative fluorescence readings and parasitemia percentages of *B*. *bovis*, *B*. *bigemina*, *T*. *equi*, and *B*. *caballi* pRBCs with different HCTs: 2.5%, 5%, and 10%. (A) *B*. *bovis* pRBCs. (B) *B*. *bigemina* pRBCs. (C) T. *equi* pRBCs. (D) B. *caballi* pRBCs. Each value is presented as the mean of triplicates after subtraction of the background fluorescence for non-parasitized RBCs.

The correlation between the fluorescence values and microscopy values was assessed in this assay using three different HCTs (2.5%, 5%, and 10%). Correlating relative fluorescence units (RFUs) with parasitemias for bovine *Babesia* parasites showed significant linear relationships with R^2^ values of 0.9945, 0.9328, and 0.9930 for *B*. *bovis* and R^2^ values of 0.9842, 0.9637, and 0.9387 for *B*. *bigemina* with HCTs of 2.5%, 5%, and 10%, respectively (Fig 1A and 1B). For equine *Babesia* and *Theileria* parasites, significant linear relationships between the fluorescence and parasitemia values were only seen for 5% and 10% HCTs with R2 values of 0.9764 and 0.9946 for *T*. *equi* and R^2^ values of 0.9786 and 0.9315 for *B*. *caballi* (Fig 1C and 1D). In summary, strong correlations (R^2^ > 0.97) were obtained at 2.5% and 10% HCTs for *B*. *bovis*, 2.5% HCT for *B*. *bigemina*, 5% and 10% HCTs for *T*. *equi*, and 5% HCT for *B*. *caballi*.

### Validation of modified high-throughput screening (HTS) assays

To evaluate the quality of the assay, the Z’ factor, S/N ratio, % CV_max_, and % CV_min_ were calculated for each of the three HCTs (2.5%, 5%, and 10%). For bovine *Babesia* parasites, the Z’ factors were similar for all HCTs within a range of 0.60 to 0.97 (S1 Table). On the other hand, S/N ratios showed the highest values at 2.5% HCT (S1 Table). Regarding the equine parasites, the Z' factor values were greater than 0.5 at 5% HCT but lower than 0.5 for the other two HCTs (S1 Table). In addition, S/N ratios had the highest values at 5% HCT for both parasites. These results reveal the robustness and accuracy of the fluorescence-based assay with 2.5% and 5% HCTs without daily replacement of the medium for bovine and equine parasites, respectively.

### Optimization of the fluorescence-based assay

**Fig 2:**
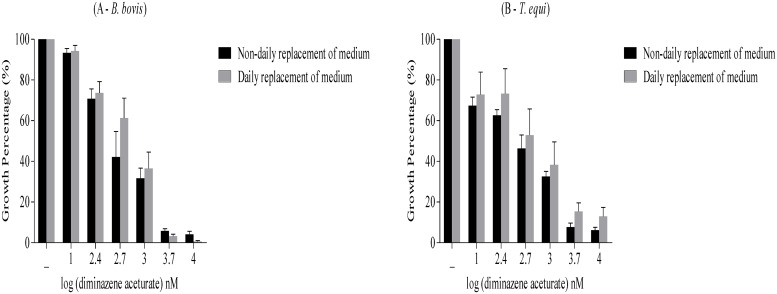
Growth inhibition of *B*. *bovis* and *T*. *equi* by diminazene aceturate estimated by the fluorescence-based method without daily replacement of the medium (black) and with daily replacement of the medium (gray). (A) Growth inhibition of *B*. *bovis* (y-axis) and log concentration of diminazene aceturate (nM) (x-axis). (B) Growth inhibition of *T*. *equi* (y-axis) and log concentration of diminazene aceturate (nM) (x-axis). Each value is presented as the mean of three triplicate wells ± SD after subtraction of the background fluorescence for non-parasitized RBCs.

Optimization of the fluorescence-based assay required determination of the IC_50_ values of diminazene aceturate for *B*. *bovis* and *T*. *equi* by fluorescence- and microscopy-based methods in cultured parasites after 4 days of treatment without daily replacement of the medium at the optimal HCTs (2.5% and 5% for *B*. *bovis* and *T*. *equi*, respectively) in three separate experiments. The results obtained by the two assays at 2.5% and 5% HCTs, for *B*. *bovis and T*. *equi*, respectively were not significantly different (Table 1). Next, to exclude the death of parasites due to the non-daily replacement of the medium, the same experiment was applied with 2.5% HCT for *B*. *bovis* and 5% HCT for *T*. *equi* with daily replacement of the medium, and the IC_50_ value was calculated by the fluorescence-based method. The results revealed no significant difference between the IC_50_ values obtained from the experiment with daily replacement of the medium and that without daily replacement of the medium (Table 2; Fig 2A and 2B). The absolute values obtained from the fluorescence—based assay after background subtraction of uninfected bovine RBCs for both cultures of *B*. *bovis* are also reported (S4A Fig). These results revealed the usefulness of the fluorescence-based assay for mass drug screenings in *in vitro* cultures of bovine *Babesia* parasites and equine *Babesia* and *Theileria* parasites with 2.5% and 5% HCTs, respectively, without daily replacement of the medium.

**Table 1:** IC_50_ values of the diminazene aceturate drug evaluated for *B*. *bovis* and *T*. *equi* by fluorescence- and microscopy-based methods without daily replacement of the medium with the optimal HCTs.

Organism	HCT (%)	IC_50_ values (nM)^a^		*P* value^b^
		Fluorescence-based method	Microscopy	
***B*. *bovis***	2.5	450 ± 90	517 ± 2	0.26
***T*. *equi***	5	540 ± 50	660 ± 280	0.50

^a^ IC_50_ values for each HCT were calculated on the fourth day based on the growth inhibitions determined by fluorescence- and microscopy-based methods in three separate experiments. Each drug concentration was made in triplicate in each experiment, and the final obtained IC_50_s were the mean of three obtained values from separate experiments.

^b^ The differences between IC_50_ values calculated by fluorescence- and microscopy-based methods for the optimal HCTs.

**Table 2:** IC_50_ values of the diminazene aceturate drug evaluated for *B*. *bovis* and *T*. *equi* with and without daily replacement of the medium using the fluorescence-based method.

Organism	IC_50_ values (nM)^a^		*P* value^b^
	Without daily replacement of the medium	With daily replacement of the medium	
***B*. *bovis***	450 ± 90	720 ± 220	0.12
***T*. *equi***	540 ± 50	610 ± 310	0.72

^a^ IC_50_ values for diminazene aceturate were determined on the fourth day of *in vitro* culture using the fluorescence-based method with 2.5% and 5% HCTs for *B*. *bovis* and *T*. *equi*, respectively, in three separate experiments. Each drug concentration was made in triplicate in each experiment, and the final obtained IC_50_s were the mean of three obtained values from separate experiments.

^b^ The differences between IC_50_ values determined using the fluorescence-based method with and without daily replacement of the medium were not statistically significant.

### Fluorescence-based assay for evaluation of the *in vitro* growth inhibitory effects

**Fig 3:**
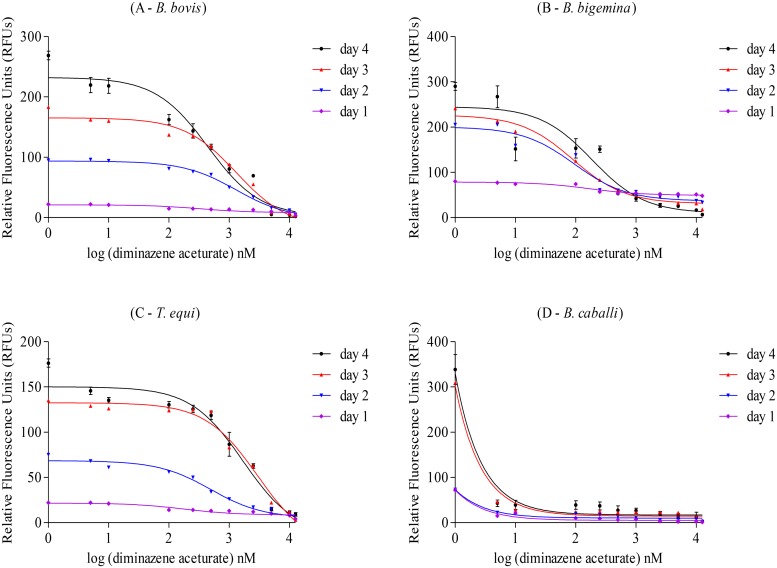
Fluorescence-based monitoring of diminazene aceturate-induced growth inhibition of *B*. *bovis*, *B*. *bigemina*, *T*. *equi*, and *B*. *caballi* during 4 days of treatment. (A) Correlation between RFUs (y-axis) and log concentration of diminazene aceturate (nM) (x-axis) on *B*. *bovis*. (B) Correlation between RFUs (y-axis) and log concentration of diminazene aceturate (nM) (x-axis) on *B*. *bigemina*. (C) Correlation between RFUs (y-axis) and log concentration of diminazene aceturate (nM) (x-axis) on *T*. *equi*. (D) Correlation between RFUs (y-axis) and log concentration of diminazene aceturate (nM) (x-axis) on *B*. *caballi*. Each value represents a mean of triplicate wells after subtraction of the background fluorescence for non-parasitized RBCs.

**Fig 4:**
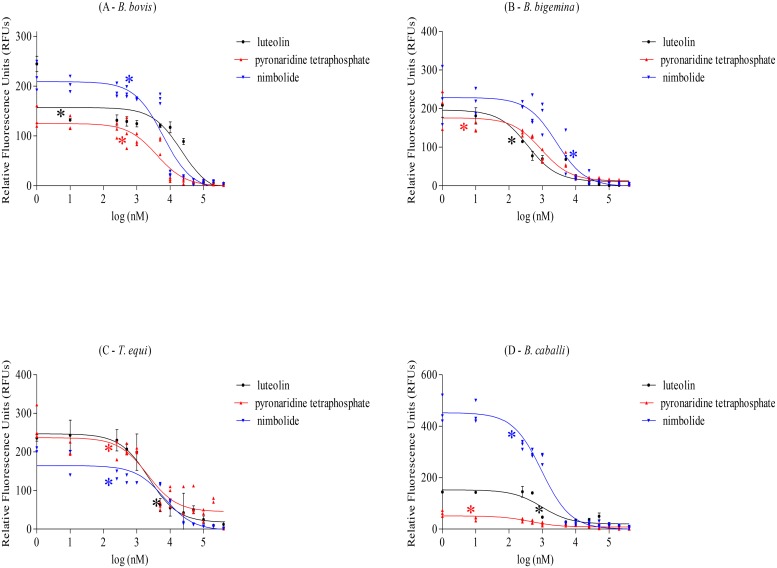
Fluorescence-based monitoring of luteolin-, pyronaridine tetraphosphate-, and nimbolide-induced growth inhibition of *B*. *bovis*, *B*. *bigemina*, *T*. *equi*, and *B*. *caballi* on the fourth day of treatment. (A) Correlation between luteolin, pyronaridine tetraphosphate, and nimbolide concentrations and RFUs on *B*. *bovis*. (B) Correlation between luteolin, pyronaridine tetraphosphate, and nimbolide concentrations and RFUs on *B*. *bigemina*. (C) Correlation between luteolin, pyronaridine tetraphosphate, and nimbolide concentrations and RFUs on *T*. *equi*. (D) Correlation between luteolin, pyronaridine tetraphosphate, and nimbolide concentrations and RFUs on *B*. *caballi*. Each value represents a mean of triplicate wells after subtraction of the background fluorescence for non-parasitized RBCs. Statistically significant differences are indicated by asterisks (*P <0.05) between the drug-treated cultures and the control cultures.

We assessed parasitemia using the fluorescence based assay in day 1 to day 4 RBC cultures infected with four hemoparasites and exposed to six drugs (Figs 3 and 4; S1 and S2 Figs). Data showed that fluorescence values were inversely correlated with drug concentrations.

The maximum parasitemias of all parasites in this study in the control cultures were observed on the fourth day. Therefore, the fluorescence values of the experiments were determined on the fourth day of treatment.

**Fig 5:**
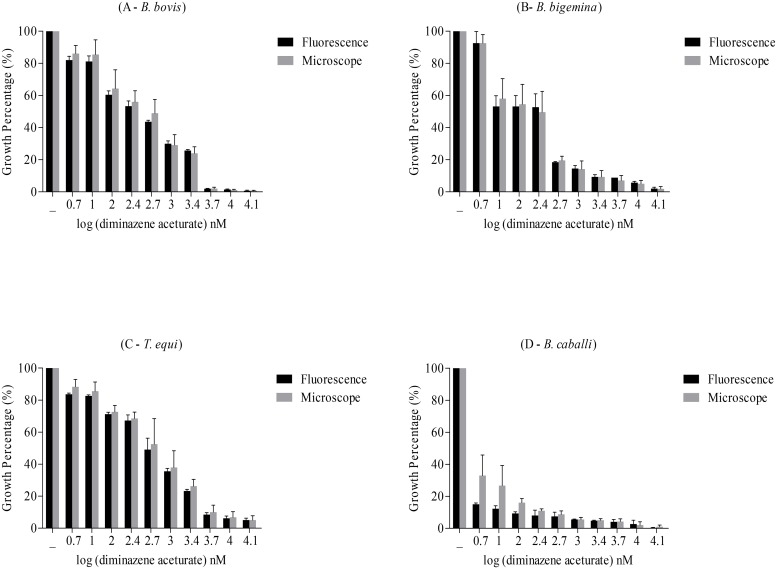
Growth inhibition of *B*. *bovis*, *B*. *bigemina*, *T*. *equi*, and *B*. *caballi* by diminazene aceturate on the fourth day estimated by the fluorescence-based method (black) and microscopy (gray). (A) Growth inhibition of *B*. *bovis* (y-axis) and log concentration of diminazene aceturate (nM) (x-axis). (B) Growth inhibition of *B*. *bigemina* (y-axis) and log concentration of diminazene aceturate (nM) (x-axis). (C) Growth inhibition of *T*. *equi* (y-axis) and log concentration of diminazene aceturate (nM) (x-axis). (D) Growth inhibition of *B*. *caballi* (y-axis) and log concentration of diminazene aceturate (nM) (x-axis). Each value is presented as the mean of three triplicate wells ± SD.

The IC_50_ values for the six drugs commonly used in the field, such as diminazene aceturate, luteolin, pyronaridine tetraphosphate, nimbolide, gedunin, and enoxacin were determined and compared to those obtained by the microscopy method. These drugs were used with the indicated concentrations: 5, 10, 100, 250, 500, 1000, 2500, 5000, 10000, and 15000 nM for diminazene aceturate; 10, 250, 500, 1000, 5000, 10000, 25000, 50000, 100000, 200000, and 400000 nM for luteolin, pyronaridine tetraphosphate, nimbolide, and gedunin; and 10, 250, 500, 1000, 5000, 10000, 25000, 50000, and 100000 nM for enoxacin. Strikingly, the IC_50_ values obtained from the current assay were significantly concordant with those obtained by microscopy (*P* > 0.05) in all tested drugs for the four parasites (Table 3; Fig 5). Additionally, the absolute values obtained from the fluorescence-based assay after background subtraction of uninfected bovine RBCs and that obtained by the microscopy method for the culture of *B*. *bovis* without daily replacement of the medium are also reported (S4B and S4C Fig). These results confirmed the successful application of our fluorescence-based assay for mass drug screenings against bovine and equine *Babesia* and *Theileria* parasites in *in vitro* cultures.

**Table 3:** IC_50_ values of antibabesial drugs evaluated for bovine *Babesia* and equine *Babesia* and *Theileria* parasites using fluorescence- and microscopy-based methods without daily replacement of the medium with 2.5% and 5% HCTs, respectively.

Drugs	*B*. *bovis*			*B*. *bigemina*			*T*. *equi*			*B*. *caballi*		
	IC_50_ values (nM)^a^			IC_50_ values (nM)^a^			IC_50_ values (nM)^a^			IC_50_ values (nM)^a^		
	Fluorescence	Microscopy	*P* value^b^	Fluorescence	Microscopy	*P* value^b^	Fluorescence	Microscopy	*P* value^b^	Fluorescence	Microscopy	*P* value^b^
**Diminazene aceturate**	410 ± 30	340 ± 190	0.56	180 ± 20	200 ± 160	0.84	670 ± 90	770 ± 280	0.58	2.8 ± 2	3.5 ± 1	0.61
**Luteolin**	5200 ± 2550*	3710 ± 1360	0.42	300 ± 50*	240 ± 190	0.62	2400 ± 890*	1350 ± 690	0.18	965 ± 10 ***	780 ± 200	0.21
**Pyronaridine tetraphosphate**	4310 ± 1960*	4890 ± 2130	0.74	700 ± 190**	530 ± 350	0.50	2140 ± 540**	1660 ± 250	0.23	440 ± 110**	360 ± 180	0.54
**Nimbolide**	6170 ± 1230**	6900 ± 430	0.38	2960 ± 1280*	1380 ± 880	0.15	6710 ± 1780	4960 ± 3640	0.49	998 ± 270**	1320 ± 30	0.10
**Gedunin**	17860 ± 2120***	16830 ± 2770	0.63	19950 ± 5520**	16900 ± 3840	0.47	12400 ± 4800*	12300 ± 1500	0.97	11180 ± 480***	9800 ± 1400	0.19
**Enoxacin**	38040 ± 3900***	35130 ± 2110	0.31	18000 ± 1510***	16510 ± 570	0.18	24700 ± 7300**	25300 ± 5200	0.91	12630 ± 3040**	10870 ± 7600	0.72

^a^ IC_50_ values for each drug were calculated on the fourth day based on the growth inhibitions determined using fluorescence- and microscopy-based methods in three separate experiments. Each drug concentration was made in triplicate in each experiment, and the final obtained IC_50_s were the mean of values obtained from three separate experiments.

^b^ The differences between the IC_50_ values calculated using fluorescence- and microscopy-based methods were not statistically significant for all six drugs analyzed.

*P < 0.05 significant differences between the drug-treated and diminazene aceturate groups.

**P ≤ 0.01 very significant differences between the drug-treated and diminazene aceturate groups.

***P ≤ 0.0001 extremely significant differences between the drug-treated and diminazene aceturate groups.

The *in vitro* growth of *B*. *bovis* was significantly inhibited (*P* < 0.05) by 10, 1000, 1000, 500 and 50000 nM luteolin, pyronaridine tetraphosphate, nimbolide, gedunin, and enoxacin, respectively (Fig 4A; S1D and S1E Fig); while, *B*. *bigemina* significantly inhibited by the same drugs at 250, 10, 5000, 1000 and 25000 nM, respectively (Fig 4B; S2D and S2E Fig). In addition, 5000, 250, 250, 25000 and 1000 nM of luteolin, pyronaridine tetraphosphate, nimbolide, gedunin and enoxacin treatments significantly inhibited the growth of *T*. *equi*, respectively (Fig 4C and S3A Fig). Finally, the *in vitro* growth of *B*. *caballi* was significantly inhibited at 1000, 10, 250, 10000 and 1000 nM luteolin, pyronaridine tetraphosphate, nimbolide, gedunin and enoxacin treatments, respectively (Fig 4D and S3B Fig).

To determine the best effective drug against the *in vitro* growth of bovine and equine hemoparasites and examine whether IC_50_ values of compounds differ from each other, ranking of drug potencies were assessed. The IC_50_ of the diminazene aceturate determined by the fluorescence-based assay of each parasite was used as a control. The obtained IC_50_s from all treated groups showed different levels of statistical significant differences (*P* < 0.05) in comparison with diminazene aceturate treated group *in vitro* cultures of four parasites (Table 3). For *B*. *bovis*, luteolin and pyronaridine tetraphosphate treated groups showed the lowest statistical significant difference, followed by nimbolide, gedunin and enoxacin (Table 3). For *B*. *bigemina*, luteolin and nimbolide treated groups showed the lowest statistical difference in comparison with diminazene aceturate treated group followed by pyronaridine tetraphosphate, gedunin and enoxacin (Table 3). While, luteolin and gedunin showed the lowest statistical significant difference in comparison with diminazene aceturate treated group *in vitro* culture of *T*. *equi*, followed by pyronaridine tetraphosphate, nimbolide and enoxacin (Table 3). Finally, for *B*. *caballi*, pyronaridine tetraphosphate, nimbolide and enoxacin showed very statistical significant difference in comparison with diminazene aceturate treated group, while, other treated groups, exhibited an extremely significant difference (Table 3). These results suggest that luteolin has the best activity against the *in vitro* growth of bovine *Babesia* and *T*. *equi* parasites next to diminazene aceturate. While, for *B*. *caballi* pyronaridine tetraphosphate showed the best activity after diminazene aceturate. Other drugs including, nimbolide, gedunin and enoxacin were effective but with lower activity against the *in vitro* growth of bovine and equine hemoparasites.

## Discussion

In recent years, antibabesial drugs commonly used in field, such as diminazene aceturate and imidocarb dipropionate [21], have shown some drawbacks, including toxicity and resistance [5]. Therefore, developing safer antibabesial medicaments merits priority in veterinary treatment research. However, several studies regarding the mode of action and target of new candidates that require more time, effort, and money should be applied. Therefore, the quest for future babesiosis drugs should be shifted to the *in vitro* screening of large-scale chemicals either commercially available or non-commercially available compounds. Then, the compounds that show potent antibabesial effects *in vitro* should be examined for their inhibitory effects *in vivo* before application in the field. In the current study, we attempted to establish an assay using SGI stain suitable for mass drug screening *in vitro* for *B*. *bovis*, *B*. *bigemina*, *B*. *caballi*, and *T*. *equi*.

The fluorescence-based assay has been approved for *in vitro* evaluation and drug screening for certain hemoprotozoan parasites [8]. The assay depends on DNA detection using SGI stain. This stain is an asymmetrical cyanine dye that has been used as a substitute for ethidium bromide in molecular biology for several years. Its mode of action depends on the interaction with double-stranded DNA, preferring G and C base pairs [22]. This interaction yields a highly fluorescent, absorbing light at a wavelength between 390 and 505 nm, with a peak at 497 nm and a secondary peak near 254 nm. It emits light at 505 to 615 nm, with a peak at 520 nm [http://www.sigmaaldrich.com/sigma-aldrich/datasheet/s9430dat.pdf]. The assay quality is determined by statistical parameters including the Z’-factor, S/N ratio, % CV_max_, and % CV_min_ [19, 23, 24]. The Z’-factor represents the separation of the distributions between the positive and negative controls, and its value should be ≥ 0.5 [19]. Low values of the Z’ factor indicate that the assay format is not effective for generating useful data. However, higher values of the S/N ratio and % CV_min_ revealed the strength and accuracy of the assay in generating reliable data [19].

In the current study, we evaluated the usefulness of our modified fluorescence-based assay for mass drug screening for *Babesia* and *Theileria* parasites. The modifications, including the final volume of the medium, hematocrit, and the non-daily replacement of the medium, improved the performance of the assay and made it more practical for the large-scale screening of drugs. The results of the HTS assay for *B*. *bovis* with 2.5% HCT generated S/N ratios 1–10 and 12–203 times higher than those obtained from our previous laboratory assay [10] and those reported in malaria research [11], respectively. Meanwhile, the S/N ratios obtained from the assay with 5% HCT for equine *Babesia* and *Theileria* parasites were 6–16 times higher than those obtained in malaria research [11].

Furthermore, the IC_50_ values obtained from the fluorescence assay without daily replacement of medium at 2.5% and 5% HCTs for bovine *Babesia* and equine *Babesia* and *Theileria* parasites, respectively, for diminazene aceturate, luteolin, pyronaridine tetraphosphate, nimbolide, gedunin, and enoxacin were similar to those obtained by microscopy in our study and those obtained in previous reports [10, 20, 25]. Taken together, the consistency in IC_50_ values calculated by these two methods highlights the robustness of the current assay as an accurate, simple, and rapid method for mass drug screening in an *in vitro* culture.

The drugs luteolin, pyronaridine tetraphosphate, and enoxacin had not yet been tested against *Babesia* and *Theileria* species but have been previously documented to be effective against the growth of *Plasmodium falciparum* [26, 27, 28]. Nimbolide was previously used only against *B*. *bovis* [10], but its inhibitory effect against other *Babesia* and *Theileria* parasites had not yet been evaluated. The IC_50_ values of nimbolide for *B*. *bovis* and *T*. *equi* parasites were higher than the value calculated for *P*. *falciparum* 2000 nM (0.95 microgram/ml) [29]. In contrast, the IC_50_ values of nimbolide for *B*. *bigemina* and *B*. *caballi* parasites were, respectively, similar to and lower than that calculated for *P*. *falciparum* [29]. Luteolin is one of the flavonoids present in fruits, vegetables, wine, tea, and coffee [30]. In addition, this plant product has antioxidant, antitumor, anti-inflammatory, antimicrobial, antiviral, and antiprotozoal activities against *Toxoplasma* [31], *Trypanosoma* [32], *Leishmania* [33], and malaria [26]. The IC_50_ values of luteolin against the growth of 3D7 and 7G8 *P*. *falciparum* have been reported to be 11000 ± 1000 nM and 12000 ± 1000 nM, respectively [26]. These values appeared to be higher than those for bovine and equine *Babesia* and equine *Theileria* parasites determined by the current assay, which might indicate the susceptibility of *Babesia* parasites to this drug. Pyronaridine tetraphosphate is a Chinese drug that has a strong inhibitory effect against chloroquine-sensitive and-resistant strains of *P*. *falciparum* [34, 35]. The IC_50_ value of pyronaridine has been reported to be 2 nM against the growth of KT1 and KT3 *P*. *falciparum* [27], which is lower than the values obtained for *Babesia* and equine *Theileria* parasites in the current study. Enoxacin is one of the fluoroquinolones, a potent DNA gyrase inhibitor that had the lowest IC_50_ values against FCC1 and VNS strains of *P*. *falciparum* [28, 36]. The IC_50_ values of enoxacin for *Babesia* and *Theileria* parasites were significantly higher than the IC_50_ values for *P*. *falciparum*, which ranged from 2300 to 4400 nM [28, 37]. Finally, the obtained IC_50_ values of diminazene aceturate for *B*. *caballi* were lower than and nearly similar to those previously calculated by microscopy [38, 20], respectively.

In summary, our optimized fluorescence-based assay with 2.5% and 5% HCTs without daily replacement of the medium for bovine and equine *Babesia* and *Theileria* parasites, respectively, offers a new approach for accurate, simple, and rapid detection of *Babesia* and *Theileria* parasites and large-scale screening of antibabesial drugs in an *in vitro* culture. In addition, luteolin and pyronaridine tetraphosphate drugs exhibited the best growth inhibition of bovine and equine hemoparasites next to diminazene aceturate, fellowed by nimbolide, gedunin and enoxacin. These drugs might be more effective if used as part of a combination therapy rather than a single therapy. Further studies are required for analyzing the synergistic or an antagonistic effect of these drugs when used in combination with each other, and to determine the best effective composition ratio for the growth inhibition of bovine and equine hemoparasites for clinical application.

## Supporting Information

S1 Fig Fluorescence-based monitoring of luteolin, pyronaridine tetraphosphate, nimbolide, gedunin and enoxacin-induced growth inhibition of *B*. *bovis* during 4 days of treatment without daily replacement of medium. (A) Fluorescence-based monitoring of luteolin-induced growth inhibition of *B*. *bovis*. (B) Fluorescence-based monitoring of pyronaridine tetraphosphate-induced growth inhibition of *B*. *bovis*. (C) Fluorescence-based monitoring of nimbolide-induced growth inhibition of *B*. *bovis*. (D) Fluorescence-based monitoring of gedunin-induced growth inhibition of *B*. *bovis*. (E) Fluorescence-based monitoring of enoxacin-induced growth inhibition of *B*. *bovis*. Statistically significant differences are indicated by asterisks (*P <0.05) between the drug-treated cultures and the control cultures. Each value represents the mean of triplicate wells after subtraction of the background fluorescence for non-parasitized RBCs.
(PDF)

S2 Fig Fluorescence-based monitoring of luteolin, pyronaridine tetraphosphate, nimbolide, gedunin and enoxacin-induced growth inhibition *B*. *bigemina* during 4 days of treatment without daily replacement of medium. (A) Fluorescence-based monitoring of luteolin-induced growth inhibition of *B*. *bigemina*. (B) Fluorescence-based monitoring of pyronaridine tetraphosphate-induced growth inhibition of *B*. *bigemina*. (C) Fluorescence-based monitoring of nimbolide-induced growth inhibition of *B*. *bigemina*. (D) Fluorescence-based monitoring of gedunin-induced growth inhibition of *B*. *bigemina*. (E) Fluorescence-based monitoring of enoxacin-induced growth inhibition of *B*. *bigemina*. Statistically significant differences are indicated by asterisks (*P <0.05) between the drug-treated cultures and the control cultures. Each value represents the mean of triplicate wells after subtraction of the background fluorescence for non-parasitized RBCs.
(PDF)

S3 Fig Fluorescence-based monitoring of gedunin and enoxacin-induced growth inhibition of *T*. *equi*, and *B*. *caballi* on the fourth day of treatment. (A) Correlation between gedunin and enoxacin concentrations and RFUs on *T*. *equi*. (B) Correlation between gedunin and enoxacin concentrations and RFUs on *B*. *caballi*. Each value represents a mean of triplicate wells after subtraction of the background fluorescence for non-parasitized RBCs. Statistically significant differences are indicated by asterisks (*P <0.05) between the drug-treated cultures and the control cultures.
(PDF)

S4 Fig The absolute values of growth inhibition for *B*. *bovis* by diminazene aceturate on the fourth day of treatment. (A) Growth inhibition of *B*. *bovis* (x-axis) and log concentration of diminazene aceturate (nM) (y-axis) by the fluorescence-based method without daily replacement of the medium (black) and with daily replacement of the medium (gray). (B) Growth inhibition of *B*. *bovis* by diminazene aceturate on the fourth day estimated by the fluorescence-based method. (C) Growth inhibition of *B*. *bovis* by diminazene aceturate on the fourth day estimated by the microscope-based method. Each value is presented as the mean of three triplicate wells ± SD after subtraction of the background fluorescence for non-parasitized RBCs.
(PDF)

S1 Table Statistical parameters for determining the quality of the high-throughput screening (HTS) assay in *B*. *bovis*, *B*. *bigemina*, *T*. *equi*, and *B*. *caballi* parasites with different percentages of HCTs. S/N ratio = Signal to noise, % CV_max_ = coefficient of variation at the maximum signal and % CV_min_ = coefficient of variation at the minimum signal.
(DOCX)
