# Supplementary figures and images for: Optimization of a Fluorescence-Based Assay for Large-Scale Drug Screening against Babesia and Theileria Parasites
Source: PLoS One. 2015 Apr 27;10(4):e0125276. doi: 10.1371/journal.pone.0125276 (PMC4411034; doi:10.1371/journal.pone.0125276)

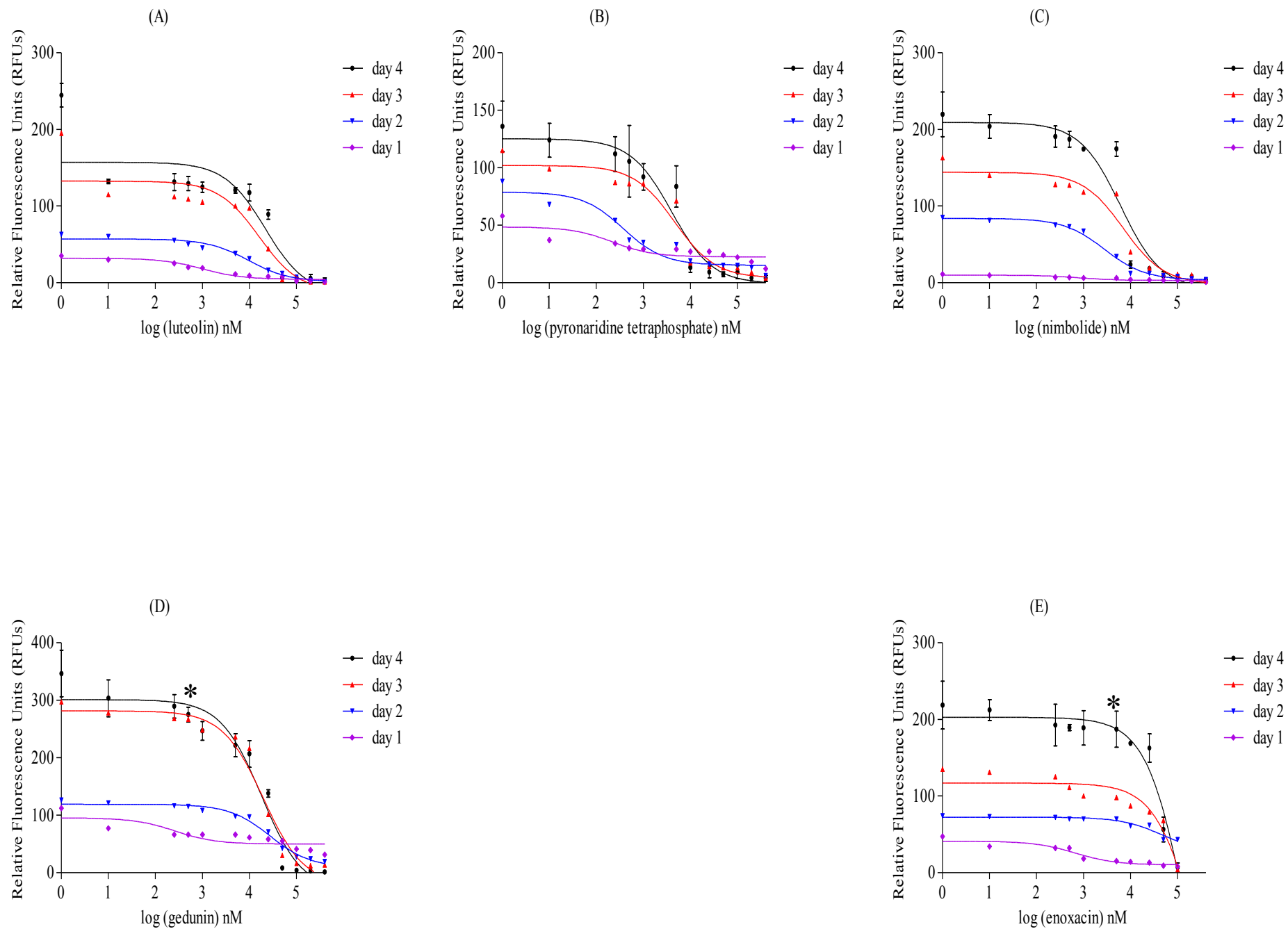

S1\_Fig.

Supplement: S1 Fig — (A) Fluorescence-based monitoring of luteolin-induced growth inhibition of B. bovis. (B) Fluorescence-based monitoring of pyronaridine tetraphosphate-induced growth inhibition of B. bovis. (C) Fluorescence-based monitoring of nimbolide-induced growth inhibition of B. bovis. (D) Fluorescence-based monitoring of gedunin-induced growth inhibition of B. bovis. (E) Fluorescence-based monitoring of enoxacin-induced growth inhibition of B. bovis. Statistically significant differences are indicated by asterisks (*P <0.05) between the drug-treated cultures and the control cultures. Each value represents the mean of triplicate wells after subtraction of the background fluorescence for non-parasitized RBCs. (PDF) [file pone.0125276.s001.pdf]

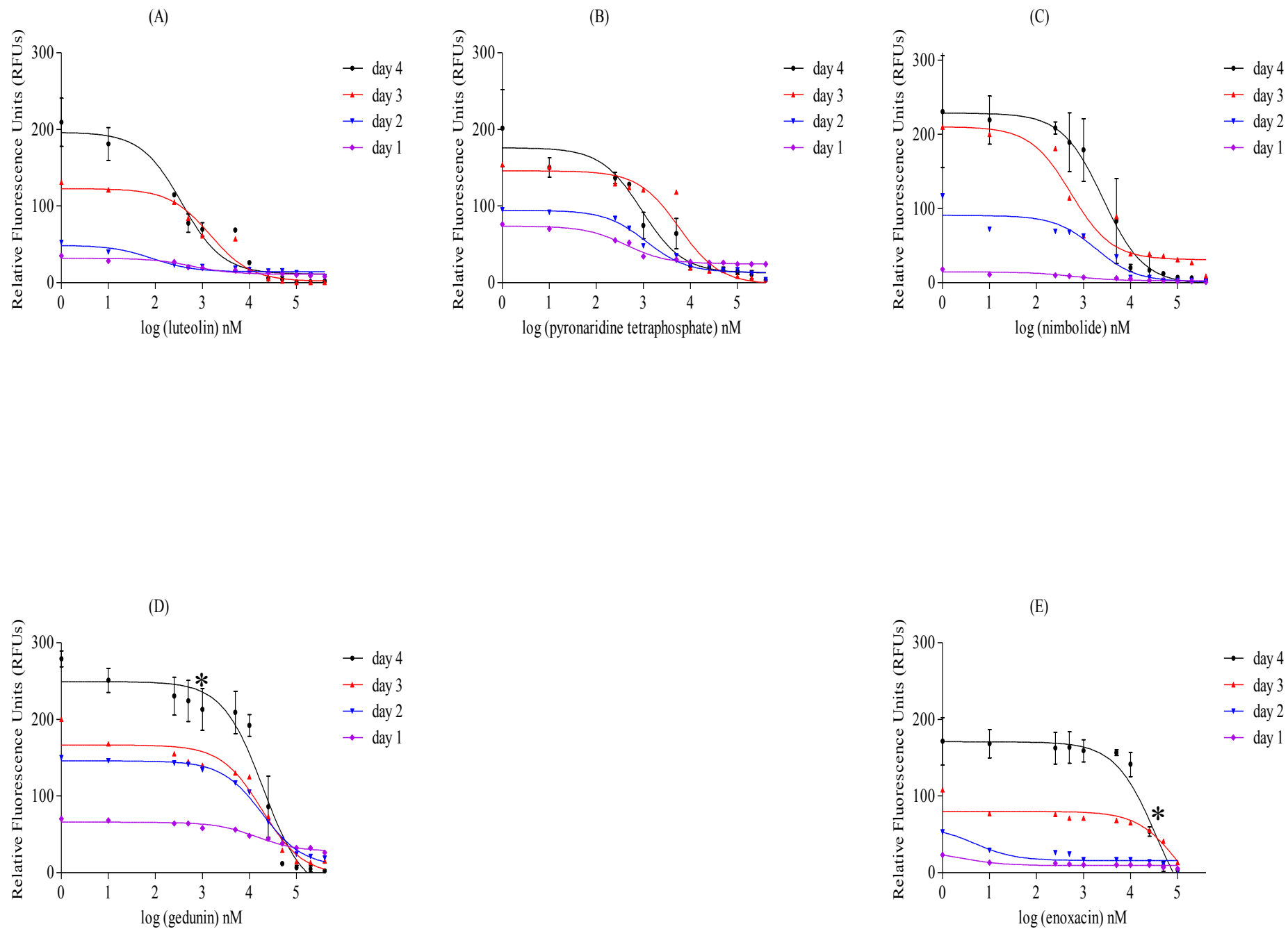

S2\_Fig.

Supplement: S2 Fig — (A) Fluorescence-based monitoring of luteolin-induced growth inhibition of B. bigemina. (B) Fluorescence-based monitoring of pyronaridine tetraphosphate-induced growth inhibition of B. bigemina. (C) Fluorescence-based monitoring of nimbolide-induced growth inhibition of B. bigemina. (D) Fluorescence-based monitoring of gedunin-induced growth inhibition of B. bigemina. (E) Fluorescence-based monitoring of enoxacin-induced growth inhibition of B. bigemina. Statistically significant differences are indicated by asterisks (*P <0.05) between the drug-treated cultures and the control cultures. Each value represents the mean of triplicate wells after subtraction of the background fluorescence for non-parasitized RBCs. (PDF) [file pone.0125276.s002.pdf]

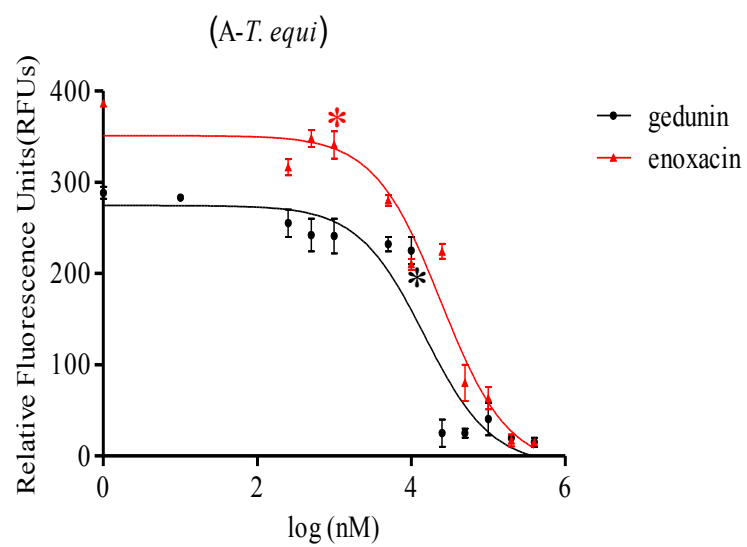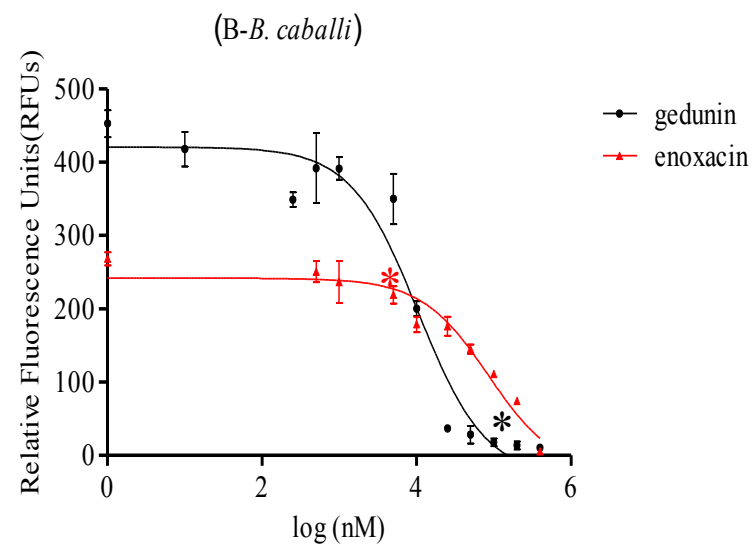

Supplement: S3 Fig — (A) Correlation between gedunin and enoxacin concentrations and RFUs on T. equi. (B) Correlation between gedunin and enoxacin concentrations and RFUs on B. caballi. Each value represents a mean of triplicate wells after subtraction of the background fluorescence for non-parasitized RBCs. Statistically significant differences are indicated by asterisks (*P <0.05) between the drug-treated cultures and the control cultures. (PDF) [file pone.0125276.s003.pdf]

(A)

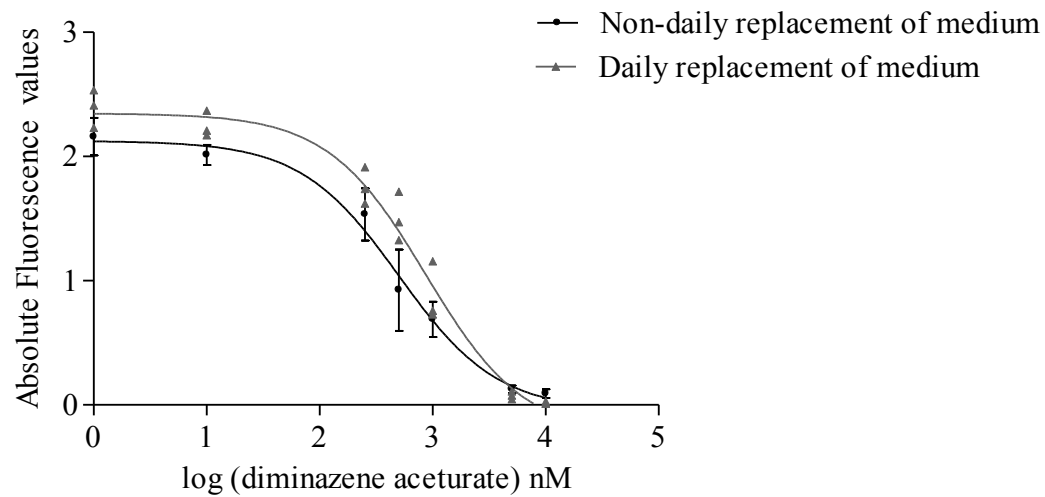

(B)

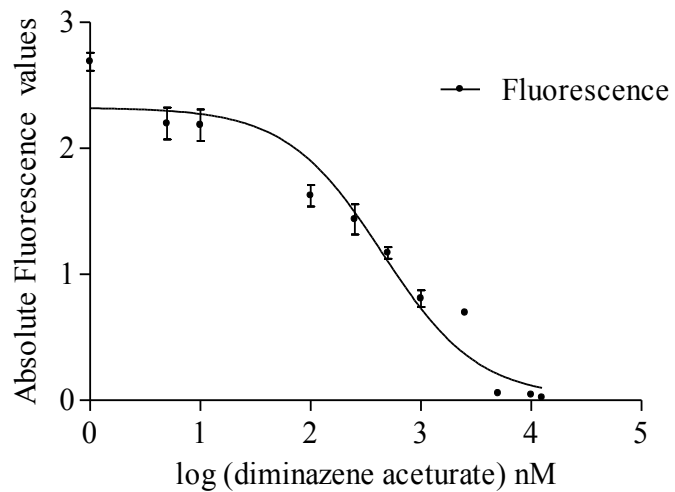

(C)

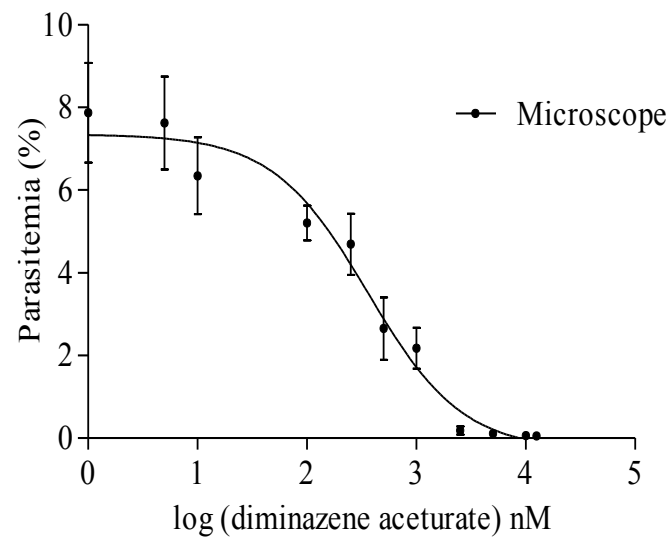

Supplement: S4 Fig — (A) Growth inhibition of B. bovis (x-axis) and log concentration of diminazene aceturate (nM) (y-axis) by the fluorescence-based method without daily replacement of the medium (black) and with daily replacement of the medium (gray). (B) Growth inhibition of B. bovis by diminazene aceturate on the fourth day estimated by the fluorescence-based method. (C) Growth inhibition of B. bovis by diminazene aceturate on the fourth day estimated by the microscope-based method. Each value is presented as the mean of three triplicate wells ± SD after subtraction of the background fluorescence for non-parasitized RBCs. (PDF) [file pone.0125276.s004.pdf]
