# Supplementary material for: Optimization of a Fluorescence-Based Assay for Large-Scale Drug Screening against Babesia and Theileria Parasites
Source: PLoS One. 2015 Apr 27;10(4):e0125276. doi: 10.1371/journal.pone.0125276 (PMC4411034; doi:10.1371/journal.pone.0125276)
Supplement: S1 Table — S/N ratio = Signal to noise, % CVmax = coefficient of variation at the maximum signal and % CVmin = coefficient of variation at the minimum signal. (DOCX) [file pone.0125276.s005.docx]

|  | *B. bovis* | | | *B. bigemina* | | | *T. equi* | | | *B. caballi* | | |
| --- | --- | --- | --- | --- | --- | --- | --- | --- | --- | --- | --- | --- |
| Parameters | HCT % | | | HCT % | | | HCT % | | | HCT % | | |
|  | **2.5** | 5 | 10 | **2.5** | 5 | 10 | 2.5 | **5** | 10 | 2.5 | **5** | 10 |
| Z’ factor | **0.69–0.74** | 0.69–0.7 | 0.72–0.97 | **0.76–0.8** | 0.60–0.81 | 0.79–0.84 | -67.5 to -1.56 | **0.84–0.85** | 0.41–0.44 | -11.6 to -24 | **0.76–0.86** | -2.28–0.36 |
| S/N ratio | **53.3–892.3** | 46.8–101.9 | 24.4–238.5 | **57.4–171.4** | 29.8–144.6 | 31.6–119.3 | 0.089–3.05 | **27.1–61.7** | 20.5–20.7 | 0.20–0.47 | **35.4–70.2** | 5.5–8.3 |
| % CV_max_ | **8.2–9.5** | 7.3–8.9 | 0.48–5.2 | **4.9–6.32** | 3–12.3 | 1.7–3.6 | 12.5–13 | **0.81–2.3** | 8.8–13.2 | 40.3–56 | **4.7–4.8** | 15.7–71.3 |
| % CV_min_ | **50–89.5** | 48.2–68.5 | 33–64.4 | **44.3–75.3** | 33.3–57.6 | 26.6–43.8 | 10.4–12.4 | **4.5–21.3** | 6.8–90.1 | 49.1–94.5 | **45.6–92.7** | 24–33.3 |
